# From Prevention To Peril: IVC Filter Strut Migration Leading to Cardiac Tamponade

**DOI:** 10.51894/001c.144623

**Published:** 2025-09-30

**Authors:** Angela Peterson

**Affiliations:** 1 Department of Emergency Medicine Henry Ford Wyandotte

## 111

### INTRODUCTION

Inferior vena cava (IVC) filter placement is a common intervention for preventing pulmonary embolism when anticoagulation is contraindicated. However, the incidence of IVC filter migration and fracture has risen to approximately 16%, correlating with increased filter use over the past four decades. Consequently, the incidence of cardiopulmonary embolization due to fractured IVC filter struts is also on the rise. This case highlights the presentation of chest pain in the emergency department (ED) secondary to an embolized IVC filter strut, resulting in myocardial injury, hemopericardium, and cardiac tamponade.

### CASE DESCRIPTION

A 52-year-old female with history of pulmonary embolism status post IVC filter presented with 24 hours of sharp chest pain, worsened by lying flat, relieved by leaning forward. Vital signs, physical exam, and electrocardiogram were unremarkable. Labs were notable for elevated troponins and a non-elevated D-dimer. Bedside echocardiogram revealed a small pericardial effusion without tamponade physiology or right heart strain. Given concern for NSTEMI or myocarditis, the patient was admitted for further evaluation. Overnight, she developed hemodynamic instability and altered mental status due to cardiac tamponade, requiring pericardial drainage of over 300 mL of blood. A CT scan revealed migration of a fractured IVC filter strut into the right ventricle, causing perforation and hemopericardium. Attempts at percutaneous removal of the strut were unsuccessful, necessitating open thoracic surgery for strut removal and myocardial repair. The patient was discharged with outpatient follow-up for removal of the remaining IVC filter.

### DISCUSSION/CONCLUSION

This case underscores the importance of maintaining a broad differential diagnosis in the ED, particularly in the context of increasing IVC filter complications. As IVC filter use rises, emergency physicians must consider migration, fracture, and embolization as potential causes of chest pain, especially in patients with a history of IVC filter placement. Early recognition of these complications is crucial in preventing life-threatening conditions such as cardiac tamponade, cardiovascular collapse, and death.

